# Treatment of Male Breast Cancer by Dual Human Epidermal Growth Factor Receptor 2 (HER2) Blockade and Response Prediction Using Novel Optical Tomography Imaging: A Case Report

**DOI:** 10.7759/cureus.1481

**Published:** 2017-07-17

**Authors:** Debasmita Saha, Susan Tannenbaum, Quing Zhu

**Affiliations:** 1 Neag Comprehensive Cancer Center, University of Connecticut Health Center; 2 Biomedical Engineering and Radiology, Washington University, St Louis, Mo

**Keywords:** male breast cancer, neoadjuvant chemotherapy, diffuse optical tomography, monitor response, pertuzumab, brca, prediction, trastuzumab

## Abstract

Male breast cancer, although rare, is on the rise. Prospective clinical trials are unlikely and current management mirrors that of post-menopausal women. Neoadjuvant chemotherapy is widely used and pathologic complete response (pCR) predicts long-term survival. The addition of dual HER2 (human epidermal growth factor receptor 2) blockade has shown the highest pCR rates; however, there is no published data of this approach in men. Also, newer monitoring tools are necessary during a neoadjuvant therapy to help personalize treatment.

Here, we describe the case of a 64-year-old man with Stage IIB (tumor size 2 to 5 cm with involvement of axillary lymph nodes), high-grade estrogen receptor, progesterone receptor, and HER2-positive invasive ductal carcinoma with a germline breast cancer susceptibility gene 1 (BRCA1) mutation who was treated in a neoadjuvant fashion with dual HER2 blockade and platinum-based chemotherapy regimen. A novel predictive tool, ultrasound-localized diffuse optical tomography, was used to monitor his progress during treatment.

## Introduction

Male breast cancer (MBC) has a reported frequency of less than 1% in the general population, with a rising incidence. However, due to the absence of prospective trials involving men, the current management mirrors treatment established for post-menopausal women and those based on a small observational retrospective analysis. The stage of breast cancer ranges from 0 to IV and is based on the tumor size (T), nodal involvement (N) and distant metastasis (M). For women with local disease, lumpectomy with sentinel lymph node biopsy remains the surgery of choice. The addition of postoperative radiotherapy has shown to improve local control and progression-free survival for those with tumors greater than 5 centimeters (cm) in greatest dimension (T3), tumor of any size with an involvement of the chest wall or skin (T4), or those with involvement of four or more axillary lymph nodes. No data exists for such an approach in men, however. Tamoxifen is considered the gold standard for adjuvant hormonal therapy for men with hormone receptor positive cancers and has shown to decrease the development of distant disease.

Risk factors for MBC are similar to those in women, including lifestyle and genetic predispositions, such as breast cancer susceptibility genes 1 and 2 (BRCA 1 and 2), tumor protein 53 (P53), or the phosphatase and tensin homolog (PTEN) mutations. It is estimated that 10% of men with breast cancer have a genetic predisposition, with BRCA2 mutation being the most common [1]. The median age of diagnosis is 60 to 70 years and almost 85 to 90% of men have invasive ductal carcinoma [2]. Approximately 90% of MBC express hormone receptors; in contrast, only 9% are human epidermal growth factor 2 (HER2) receptor amplified (positive).

Neoadjuvant chemotherapy (NAC) is widely used in the management of breast cancer where pathologic complete response rates (pCR) predict for long-term survival. The use of neoadjuvant dual HER2 blockade with trastuzumab and pertuzumab have shown the highest pCR rates in the NeoSphere [3] and TRYPHAENA (Pertuzumab, plus trastuzumab in combination with standard neoadjuvant anthracycline-containing and anthracycline-free chemotherapy regimens in patients with HER2-positive early breast cancer: a randomized phase II cardiac safety study) [4] trials. The TRYPHAENA trial led to the first ever Food and Drug Administration (FDA) approval of a drug for non-metastatic cancer, without trial data in the adjuvant setting. However, there has been no published data on the use of NAC and such novel agents in non-metastatic HER2-positive disease in men. 

Since the absence of residual tumor in the primary tumor bed after neoadjuvant therapy strongly predicts improved survival, there are many ongoing investigations in methods to monitor response and allow early treatment modification to improve outcome. Conventional methods for monitoring NAC have not provided actionable outcomes to date. Diffuse optical tomography (DOT) uses near-infrared diffused light and measures tumor angiogenesis through total, oxygenated, and deoxygenated hemoglobin content levels and has been used as a novel tool to predict and monitor response [5]. The logistic prediction model we developed utilizes tumor pretreatment pathological parameters and hemoglobin content measured before and during first three treatment cycles to predict pCR [6-7]. 

## Case presentation

**Figure 1:**
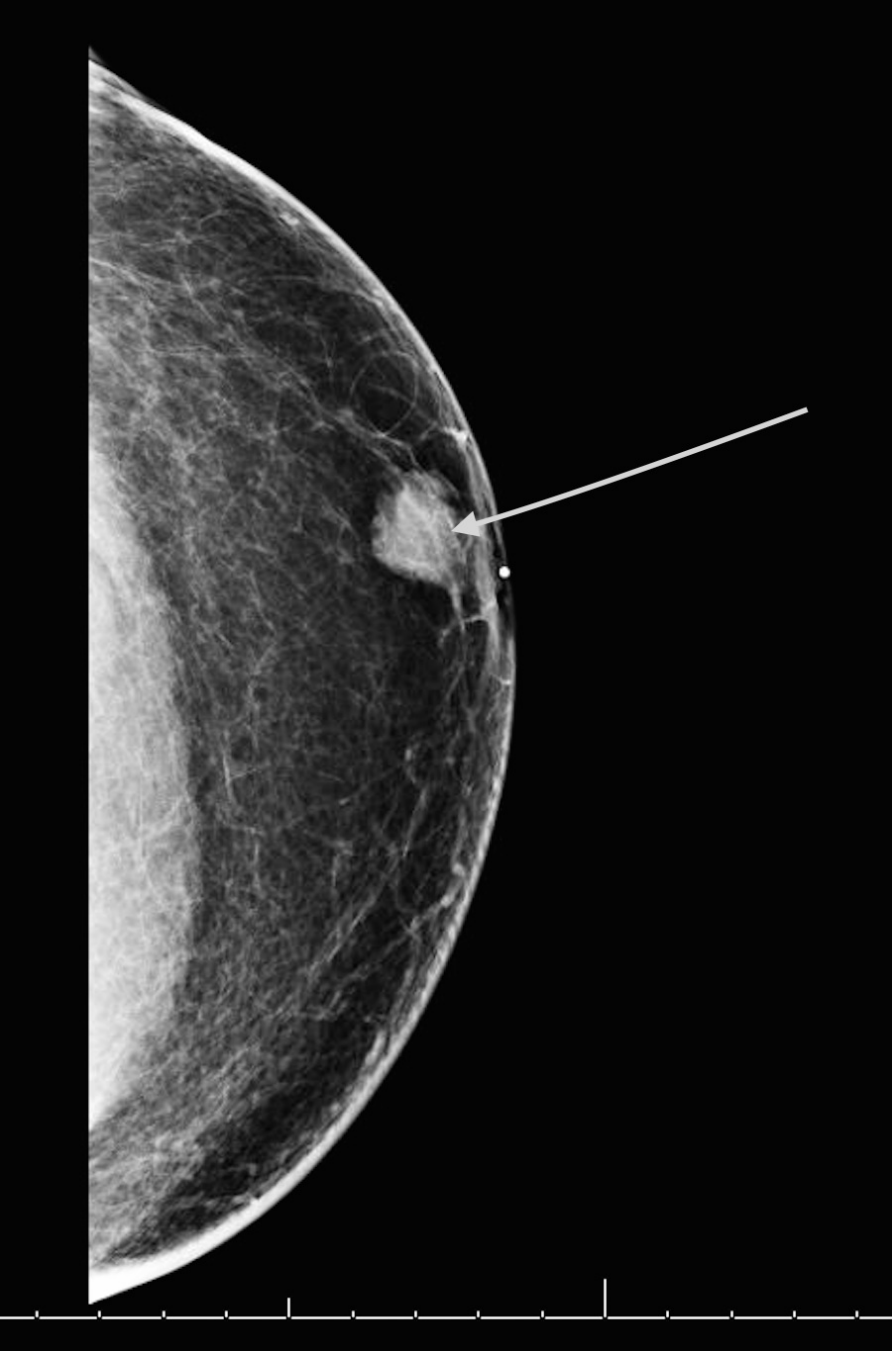
Diagnostic mammogram showing the left breast mass.

**Figure 2:**
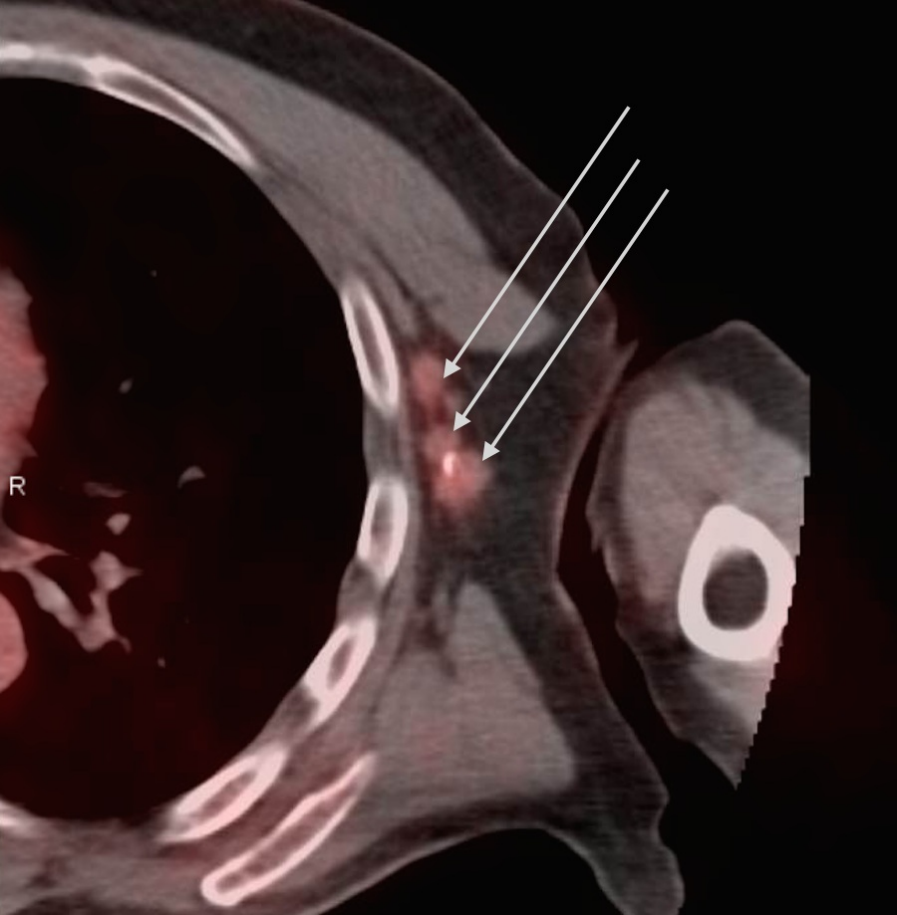
Positron emission tomography–computed tomography scan (PET-CT) showing a cluster of metabolically active left axillary lymph nodes measuring up to 1.5 cm

A 64-year-old, otherwise healthy man, presented with a painful left breast lump. He had a strong family history of breast and prostate cancers. On physical exam, he had an inverted nipple with a 2 cm firm palpable mass near the areola and numerous enlarged left axillary lymph nodes. Ultrasound (US)-guided biopsy confirmed a high-grade estrogen receptor (ER), progesterone receptor (PR), and HER2 positive invasive ductal carcinoma in the breast and axillary nodes. Staging workup with a whole body positron emission tomography–computed tomography (PET-CT) scan revealed a hypermetabolic retroareolar left breast mass and five enlarged hypermetabolic left axillary lymph nodes, consistent with his known disease. These are seen in Figures 1 and 2. Clinically, he was staged as IIB disease (tumor size 2 to 5 cm with the involvement of axillary lymph nodes). Genetic testing revealed a heterozygous germline BRCA1 mutation. Also of uncertain clinical significance were mutations in the BRCA1-interacting protein 1 (BRIP1) and postmeiotic segregation increased 2 (PMS2). 

Using the neoadjuvant approach in the setting of HER2 positive locally advanced cancer, he was given six cycles of trastuzumab, carboplatin, docetaxel, and pertuzumab (TCHP) regimen as described in Table 1.  

**Table 1:** The TCHP Regimen All drugs were given on Day 1. Repeat cycle every 21 days for 6 cycles, followed by Trastuzumab 6 mg/kg IV every 21 days to complete one year of therapy IV: intravenous; TCHP: trastuzumab, carboplatin, docetaxel and pertuzumab

TCHP Regimen:
Trastuzumab, 8 mg/kg IV for first cycle followed by 6 mg/kg IV in subsequent cycles
Pertuzumab, 840 mg IV followed by 420 mg IV
Docetaxel, 75 mg per meter square IV
Carboplatin AUC 6

**Figure 3:**
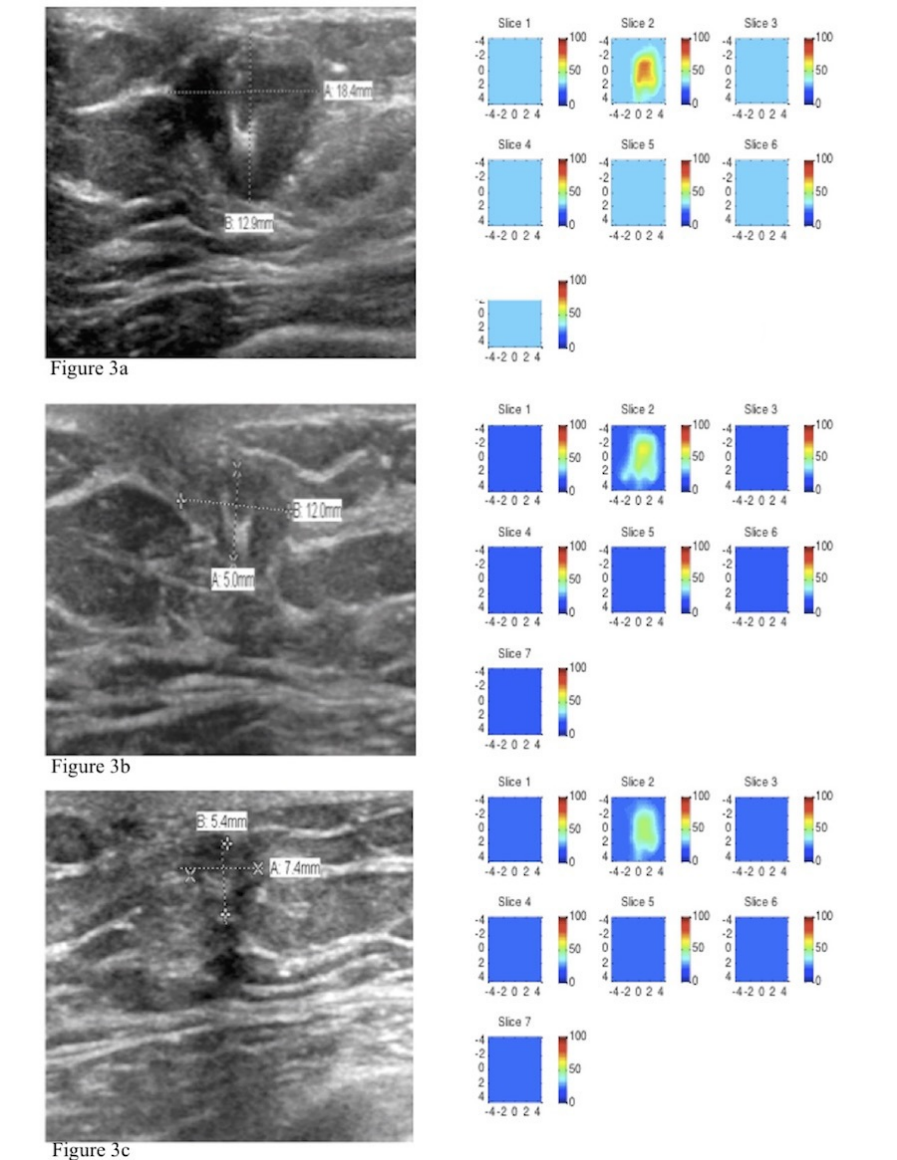
Serial ultrasounds (US) and diffuse optical tomography (DOT) total hemoglobin (tHb) maps Each figure depicts serial ultrasounds and US-DOT images taken pretreatment, at the end of three cycles of treatment, and prior to surgery. The ultrasound images are shown on the left, depicting the changes in the dimension of the tumor. US-DOT images are shown on the right with the tHb maximum levels depicted in the x and y spatial map, at depths 0.5 cm to 3.5 cm from skin to chest wall, corresponding to slices 1 to 7. The tHb levels from figures 3a, 3b, and 3c are 73, 61 and 57.5 um/L, respectively.

Ultrasound diffuse optical tomography (US-DOT) was performed before and at the end of the first three treatment cycles and again before surgery. These are shown in the series in Figure 3.  Pretreatment total hemoglobin (tHb) maximum levels measured at the end of each of the first three treatment cycles and before surgery were 73.0 micromoles per liter (umol/L), 74.9 umol/L, 72.9 umol/L, 61.0 umol/L, and 57.7 umol/L, respectively. The corresponding percentage changes as compared with the pretreatment baseline were similar for the first two treatment cycles and dropped only by 16.4% and 20.1% at the end of cycle 3 and before surgery, respectively. Using pretreatment parameters alone and with tHb levels of the first three cycles, he was predicted to be an incomplete-responder.

The University of Connecticut Health Center IRB Committee approved the study “Ultrasound and Near-Infrared Imaging for Breast Cancer Diagnosis and Neoadjuvant Chemotherapy Monitoring” (approval #12-194-6). This patient was also enrolled in that study.

He subsequently underwent a modified radical mastectomy (MRM), which revealed a 0.7 cm residual tumor within the tumor bed. Twenty lymph nodes were examined and three were positive for malignancy. Based on the Miller-Payne grading system, the overall response to neoadjuvant therapy was grade 3 (between a 30% to 90% reduction in tumor cellularity). This was consistent with what was predicted using the US-DOT. The patient opted to have a right prophylactic simple mastectomy, not a standard recommendation in male BRCA mutation carriers. He received post-mastectomy radiation and completed a full year of therapy with trastuzumab and was also started on adjuvant therapy with tamoxifen. He subsequently developed prostate cancer and he continues to be on anti-estrogen therapy as we continue to follow him closely. 

## Discussion

This case report presents a relatively uncommon case of a male with a BRCA1 gene mutation and also HER2 expression. One previous case report [8] has used a combination of trastuzumab, pertuzumab, and docetaxel in a similar setting. However, previous trials on women have demonstrated that those with BRCA mutations are especially sensitive to platinum-based chemotherapy given in the neoadjuvant setting [9]. Dual HER2 blockade in the neoadjuvant setting for HER2-positive disease is the standard of care in women. In our case, we used a combination of these approaches in the neoadjuvant setting.

During a neoadjuvant approach, it is critical to isolate the non-responders early so they can be offered an alternative treatment. No single diagnostic technique has so far been able to reliably assess pathological response to neoadjuvant therapy. Imaging techniques, such as US, mammography, and breast magnetic resonance imaging (MRI), have also been used with poor outcomes. PET-CT has shown to have a good sensitivity but a low specificity in one meta-analysis [10].

US-DOT results during treatment have shown a strong correlation with treatment outcome. This is a promising technique for monitoring the response to neoadjuvant therapy that could be used as an early predictor of nonresponders. It is used in combination with other histopathologic parameters [7], such as tumor type, Nottingham score, and receptor status. US-DOT is a functional imaging technique that is cost effective and utilizes no ionizing radiation. This method assesses the tumor vascularity from the tumor’s tHb concentration and is directly related to tumor vascular content. The change in the value of the tHb from the baseline and during the first three treatment cycles is significantly different in the groups that subsequently have a Miller-Payne grade 4 to 5 and those with grade 1 to 3. This suggests that tumors with higher vascular content respond more profoundly to systemic therapies. In the TRYPHAENA trial, 90% of the enrolled patients had clinical responses, of which almost half had a partial response; our patient fits into this subgroup of partial responders.

## Conclusions

MBC is rare and prospective clinical trials are unlikely in this population. Here, we present an unusual case of a male with an HER2-positive locally advanced disease. We used a combination of two treatment approaches, which are the standard of care in women, i.e., the use of platinum-based chemotherapy and dual HER2 blockade. This approach has not previously been described in men. Also, we demonstrated the use of a novel imaging method, which accurately predicted our patient’s incomplete response. As we continue to explore newer treatment and monitoring methods and make early changes based on our modified diagnostic and treatment protocols, further personalization of therapy will become possible, even in such uncommon presentations.
